# Late Hypersensitivity Reactions to the BNT162b2 SARS-CoV-2 Vaccine Are Linked to Delayed Skin Sensitization and Prior Exposure to Hyaluronic Acid

**DOI:** 10.3390/life12122021

**Published:** 2022-12-03

**Authors:** Ramit Maoz-Segal, Ronen Shavit, Mona Iancovici Kidon, Irena Offengenden, Diti Machnes-Maayan, Yulia Lifshitz-Tunitsky, Stanley Niznik, Nancy Agmon-Levin

**Affiliations:** 1 The Clinical Immunology, Angioedema and Allergy Unit, Center for Autoimmune Diseases, Sheba Medical Center, Tel Hashomer, Ramat Gan 52621, Israel; 2 The Sackler Faculty of Medicine, Tel Aviv University, Tel Aviv 6329302, Israel

**Keywords:** COVID-19 vaccine, dermal filler, hyaluronic acid, polyethylene glycol, late hypersensitivity, BNT162b2 vaccine

## Abstract

Background: Late hypersensitivity reactions (HSRs) to the BNT162b2-vaccine have raised concerns regarding its safety, particularly as further immunizations are required. The yield of skin testing with the BNT162b2v is unclear, as well as the risk factors and outcomes of re-immunization after late HSRs. Objective: We studied a series of patients with late HSRs to BNT162b2v. Methods: Patients referred to the Sheba medical center from December 2020 to May 2021 with late HSRs to the first dose of BNT162b2 were included. HSRs were defined as late if they appeared or lasted >24 h after inoculation. We compared late HSRs to immediate HSRs that appeared within minutes–2 h after vaccination. Intradermal testing with PEG-containing medication and BNT162b2v was performed. Results: A total of 17 patients that presented with late HSRs (study group) were compared to 34 patients with immediate HSRs (control group). Delayed sensitivity to intradermal testing of the BNT162b2v was observed in 9/17 (53%) of the study group compared to 4/34 (12%) in the control group (*p* = 0.01). Former exposure to a dermal filler with hyaluronic acid was documented among 7/17 (41%) vs. 2/34 (6%) in the study and control groups, respectively, (*p* = 0.0038). All patients who presented with late HSRs were advised to receive subsequent doses of the BNT162b2v vaccine with or without concomitant medication, and all were re-immunized successfully. Conclusions: Late HSRs to BNT162b2v were linked with positive responses to intradermal testing with the vaccine and prior exposure to derma fillers with hyaluronic acid. This may elude to an immune mechanism triggered by former exposures. Although further studies are needed, late HSRs to the BNT162b2-vaccine did not prevent patients from receiving subsequent doses of the vaccines.

## 1. Introduction

The COVID-19 pandemic, also known as the coronavirus pandemic, is an ongoing global pandemic of coronavirus disease 2019 (COVID-19) caused by severe acute respiratory syndrome coronavirus 2 (SARS-CoV-2). This ‘new arrival’ virus was first identified in Wuhan, China, in December 2019. Since its first appearance, the virus has spread to other areas of Asia and later worldwide. The World Health Organization (WHO) declared the outbreak a public health emergency of international concern on 30 January 2020 and a pandemic on 11 March 2020. As of 27 October 2022, the pandemic had caused more than 629 million cases and 6.58 million confirmed deaths, making it one of the deadliest infections in modern history.

The COVID-19 pandemic accelerated the development of the new mRNA-based vaccines such as the BNT162b2 and the mRNA-1273. These vaccines were found to be safe and effective, and are widely credited for their role in reducing the severity and death caused by COVID-19. The BNT162b2v consists of a frozen suspension of nucleoside-modified messenger RNA (mod-RNA) and inactive ingredients. The major inactive ingredient is 2-[(polyethylene glycol)-2000]-N,N-ditetradecylacetamide, which is a modified polymer of ethylene glycol, also known as ALC-0159. PEG is the common abbreviation for polyethylene glycol, which refers to a chemical compound composed of repeating ethylene glycol units. The BNT162b2v received an Emergency Use Authorization (EUA) from the FDA on December 2020. On August 2021, the FDA granted the BNT162b2v COMIRNATY^®^ full approval. Soon after the accelerated development of these vaccines, the global immunization programs were initiated throughout the world, as well as in Israel, with the aim of controlling the pandemic [1,2]. Several obstacles to this initiative were apparent early, one of which was hypersensitivity reactions to these new mRNA-based vaccines. Soon after the Israeli immunization program started, reports of hypersensitivity reactions (HSRs) raised concerns regarding the safety of additional booster doses, which were required to maintain the long-term effectivity of the vaccine. Drug hypersensitivity reactions are immune-mediated reactions to a drug or vaccine. These reactions are categorized as immediate and late hypersensitivity reactions according to reaction characteristics and the timing of the reaction. The majority of late HSRs are mild; however, severe and multi-system reactions can occur. The diagnosis is mostly clinical, but skin testing can be useful as well. Late HSRs to drugs or vaccines are common in the general population and are a frequent cause of referral for evaluation in allergy clinics worldwide [3,4,5,6]. The BNT162b2 vaccine (BNT162b2v) was the first vaccine to be available for immunization against the coronavirus in Israel. At that time, the data regarding assessment of patients and evaluation of risk factors following the HSRs to the first dose of the BNT162b2v were lacking. Moreover, it was uncertain whether more doses would be required along the way as booster doses for the general population. In addition, novel virus variants, designated by the World Health Organization (WHO) as the variants of concern (VOC), prompted consensus that the mRNA boosters’ incorporation against the VOC was the only effective strategy to counter emerging strains.

Many studies were published regarding immediate-type hypersensitivity reactions to the COVID-19 vaccines, including the use of skin testing with PEGmeds [7], but the data regarding late HSRs are limited.

Hence, in this study, we investigated late HSRs to the BNT162b2v in a series of patients referred for evaluation to our tertiary medical center. We also looked at the role of performing direct skin testing with the whole vaccine, as well as PEG-containing medications (PEGmeds), in the assessment of late HSRs. Using skin tests with the vaccine was addressed in other studies as a method for evaluating cell immunity [8]. Our aim was to use skin testing as a risk stratification tool prior to further immunization. To note, in this study we address late skin hypersensitivity reactions which appeared elsewhere to the injection site, meaning other than the “COVID arm” which is an erythematous rash at the injection site which has been described before [9].

## 2. Methods

### 2.1. Patients

Subjects that experienced late HSRs to the 1st dose of BNT162b2v from December 2020 to May 2021 and were referred to our center for evaluation were included in our study group. A comparison was performed with age- and gender-matched patients in a 2:1 ratio to patients who suffered an immediate HSR to this vaccine. All patients included in this study were referred and evaluated in the Institute for Clinical Immunology, Angioedema and Allergy in the Sheba Medical Center in Israel.

The hypersensitivity reactions to the 1st dose of BNT162b2v were categorized as follows: Late HSR was categorized by a skin rash with or without mucosal manifestations that appeared at least four hours post immunization and lasted for more than twenty-two hours. In contrast, immediate HSRs to BNT162b2v were defined accordingly if they appeared minutes and up to two hours post immunization and lasted for less than 12 h. The immediate HSRs had one or more of the following symptoms: skin rashes, angioedema, respiratory symptoms and/or gastrointestinal symptoms. Other adverse reactions to immunizations, such as numbness, weakness, fever, muscle pain, etc., were excluded as non-hypersensitivity reactions. All subjects underwent allergic consultation by an allergy specialist focusing on specific characteristics of the hypersensitivity reaction: the time of the first symptoms, systemic manifestations other than skin, duration of the symptoms and any treatment if it was received. The patients reported their history of hypersensitivity reactions to drugs and vaccines, as well as other immune-mediated diseases (e.g., chronic urticaria, mastocytosis or atopic background) and former cosmetic procedures using skin fillers, namely facial injections of hyaluronic acid preparations in the past. Finally, following evaluation via interview and skin tests, the patients were immunized with the BNT162b2v in our department under supervision.

### 2.2. The Skin Test Protocol

A skin test to assess delayed sensitivity to the vaccine or its excipient was performed using intradermal (ID) skin testing with polyethylene glycol (PEG)-containing medications (PEGmeds), namely methylprednisolone acetate depot injection (Depo-Medrol^®^) at a dilution of 1:10 (4 mg/mL) and with the BNT162b2v, reconstituted on the same day, in dilution 1:100 and 1:10. We used the leftovers of the vaccine used in our center on the same day to prepare the dilatants for skin tests to avoid the wasting of vaccine materials.

*Delayed skin sensitization* to the ID skin test was read at 24 and 48 h after the skin test via a follow-up visit or photographs sent by the patients. *Delayed skin sensitization* was confirmed if an induration greater than 3mm was evident, either to the Depo-Medrol in dilution of 1:10 or to the BNT162b2 vaccine in dilution 1:100, whereas a skin response only to the 1:10 dilution of the BNT162b2v was considered as a possible irritant and was not included in our analysis. The skin tests were conducted at least twenty-one days following resolution of the late HSRs, in order to avoid false negative tests due to treatments with anti-histamines and/or corticosteroids following the hypersensitivity reaction. Skin tests for evaluation of immediate HSRs were more extensive and included additional skin prick tests (SPTs) to the same reagents and other PEG meds as published elsewhere [10].

All skin tests were assessed by an allergy specialist.

**Statistics**

Statistical analysis was performed using IBM SPSS statistics software for Windows, version 27, 2020. The software sourced from Sheba medical center Ramat Gan, Israel. The methods used were analysis of variance (ANOVA), *t* test and/or Fisher’s exact, as appropriate. *p* values of less than 0.05 were considered significant.

## 3. Results

### 3.1. Patients’ Demographics and Clinical Backgrounds

Our cohort included seventeen patients with late HSRs to the first dose of the BNT162b2v. They were mostly females, 15/17 (88%), with a mean age of 55 ± 16 years, that were compared to thirty-four age- and gender-matched patients with immediate HSRs. Clinical manifestation of late HSRs to BNT162b2v. included generalized urticarial rash and swelling of the skin mostly located on the face. Late reactions started at least four hours post immunization and lasted for up to five days. All patients were treated with anti-histamines (100%) and 4/17 patients (23.5%) received systemic glucocorticoids, usually in the form of oral prednisone, following the late HSRs.

Comparison between the late and immediate HSRs patient groups in a 2:1 ratio revealed no differences between the groups other than former exposure to skin filler with hyaluronic acid (HA) that was significantly more common among the patients with late HSRs (*p* = 0.004) (Table 1).

### 3.2. Skin Tests

**Figure 1:**
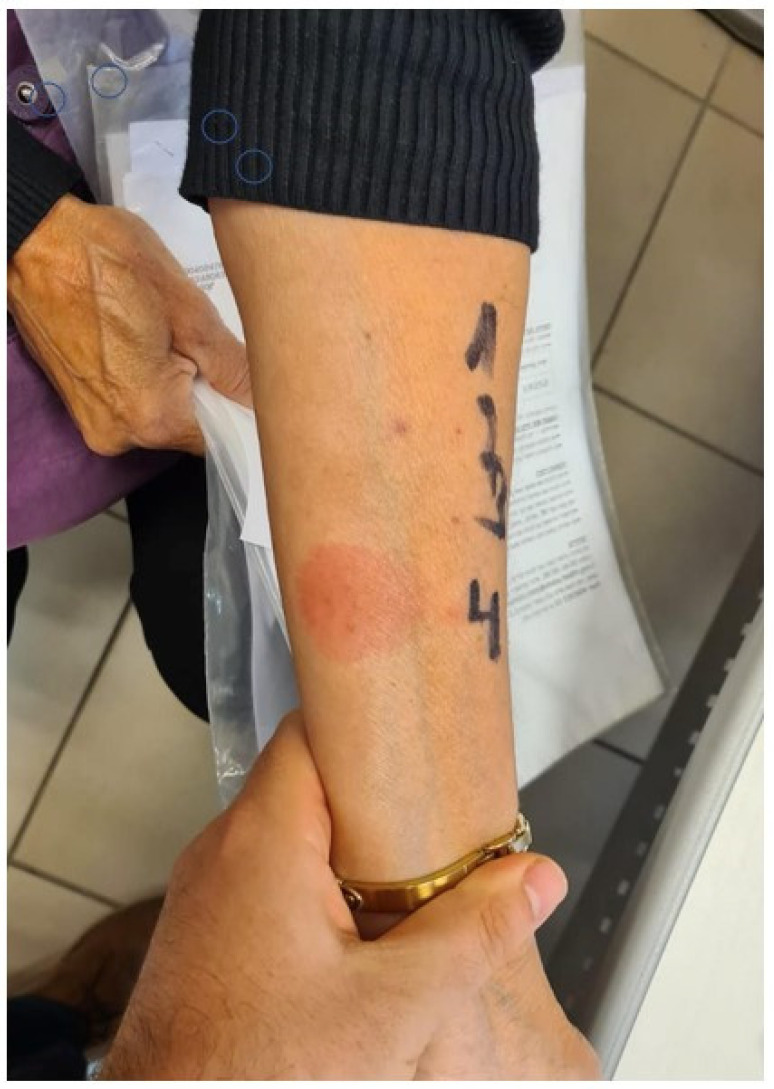
An example of a positive intradermal skin test with BNT162b2v. (1)–(3) Intradermal tests with PEGmeds, (4) intradermal test with BNT162b2v. in dilution of 1:100.

Delayed skin sensitization via direct intradermal injection with the BNT162b2v in dilution 1:100 was significantly more prevalent among the late HSRs group, 9/17 (53%), compared to 4/34 (12%) in those with immediate HSRs (*p* = 0.003). An example of a positive skin test is presented in Figure 1. None of our patients in either group showed skin sensitization via indirect assessment with the PEG meds used in this study. Former exposure to hyaluronic acid was reported by thirteen patients in our cohort: nine of them exhibited late HSRs and four exhibited immediate HSRs. Looking at the delayed skin sensitization to intradermal injection of the BNT162b2v among the patients with former exposure to hyaluronic acid revealed no statistically significant difference between the two groups: 6/9 (67%) patients with a late HSR and 2/4 (50%) patients with an immediate HSR.

### 3.3. Further Immunization with Second and Third Doses of the BNT162b2v

All 17 patients with late HSRs were advised to receive the additional doses of vaccines regardless of the skin test results. All patients were immunized with the second and third dose of the BNT162b2 vaccine according to the recommendation of the Israeli Ministry of Health. All patients were re-immunized under supervision in our allergy clinic with the supervision of an allergy specialist up to 12 h post immunization. The addition of anti-histamines one hour prior to the immunization (as pre-medication) and daily for 3–5 days following immunization was advised to all patients, according to the original reaction. No significant reactions were reported following subsequent immunizations, although 2/17 (12%) patients had a rash of which 1 required additional doses of systemic glucocorticoids. The patients were advised to contact the clinic via e-mail or phone in cases of worsening symptoms at home. None of the patients contacted the clinic as there were no symptoms at home following re-immunization with the second and third doses of the vaccine. To the best of our knowledge, there were no severe cutaneous adverse reactions (SCARs).

On the left side of the image—intradermal (ID) BNT162b2 in dilutions 1:100 and 1:10. On the right side of the image—skin prick test and ID skin tests with PEG-containing medications: (1) Normalax SPT, (2) Depo-Medrol SPT and ID, (3) Meroken SPT, (4) the vaccine SPT, (H) histamine control, (S) saline negative control.

## 4. Discussion

In this study we describe a cohort of seventeen patients with late HSRs to the BNT162b2 vaccine. The late HSRs were more prevalent among women, as expected based on previously described reports of higher prevalence of HSRs to medications and vaccines among women in general and in particular to the relatively new m-RNA vaccines [11,12,13]. Notably, all the late HSRs to the first dose of the vaccine were mild, involving mostly the skin. By October 2021, all of the seventeen patients successfully received subsequent immunizations with the second and third doses of the BNT162b2v, regardless of their skin test results.

Our results are similar to several previous reports of safely vaccinating patients with additional doses of the BNT162b2v as well as other anti-COVID vaccines following late HSRs [14,15,16,17,18,19].

In our cohort, all patients received concomitant medication (namely, pre-medication and post medications with anti-histamines) which may have contributed to the relatively low rate (12%) of HSR recurrence following booster doses, compared to the outcomes published by Pitlick et al. [14] in which only 2/10 patients received concomitant medication with a relatively high percentage, 4/10 (40%), of repeated hypersensitivity reactions after re-immunization.

Hyaluronic acid (HA, Hyaluronan, Hyaluronate) is an anionic, non-sulfated glycosaminoglycan. HA is one of the most widely used polysaccharide biopolymers in biomedical research. Application includes drug delivery, hydrogel, tissue engineering, regenerative medicine, implants, contact lenses and more. HA-based injectable filling agents are commonly used for noninvasive dermatological procedures for the correction of age-related soft tissue defects. Currently, there are many HA-based products available for use. The performance of crosslinked skin fillers depends on the HA concentration, degree of crosslinking, elastic modulus, cohesivity and type of crosslinking agent employed. PEG crosslinked with HA displays improved elasticity and more resistance to degradation. Different HA fillers’ preparations demonstrate high- and low-molecular-weight hyaluronans, achieving high concentration and low viscosity with optimal tissue diffusion which assist physicians in selecting the most appropriate filler for specific uses. In this study we demonstrated delayed skin sensitization using direct assessment with intradermal (ID) skin testing with the diluted vaccine. We saw a correlation between late skin sensitization and late HSRs to the vaccine. However, indirect assessment of skin sensitivity with PEG-containing medication was inconclusive in our study, as previously reported by Pitlick et al. [14]. Although intradermal testing was found to be of little clinical significance as all of the patients were re-immunized safety soon after regardless of the testing results, it may be of importance in order to reveal the potential mechanisms of the late HSRs. Understanding the mechanisms of such reactions is important, especially due to the fact that more doses of these vaccines will be required to maintain vaccine efficacy.

We found that prior exposures to skin fillers with hyaluronic acid (HA) were related to late skin sensitization as well as the occurrence of late HSRs to the BNT162b2v. The correlation between HA and late HSRs to mRNA vaccines was previously suggested and reported with both mRNA vaccines (BNT162b2v and mRNA-1273v) [20,21,22,23]. Currently, transient local reactions following HA injections are referred to as “delayed inflammatory reactions” with incidence of 1.1% reported per year [24]. These inflammatory reactions may occur from a few months and up to several years following HA injections [25,26]. In the particular case of the BNT162b2v, the link to a former exposure to HA via injection was suspected by the medical staff as one of the female patients in our cohort reported skin swelling a few hours post immunization located in the same locations on her face that were previously injected with HA. Similar findings were found in other publications reporting hypersensitivity reactions, including redness and face swelling at the location of former HA injections. Local HSRs to BNT162b2v were also reported at sites other than the face, such as breasts in two women following breast augmentation which occurred seventeen months and five years before immunization with the BNT162b2v [27].

The exact mechanisms of HA-related late HSRs are not known. Possible mechanisms that were suggested include current infection, local trauma and the properties of the fillers, as well as injection technique in the hand of the performer, the injection location and multiple injections. Indeed, vaccines, just like infectious agents, may induce an unintended immune adverse response in susceptible individuals. The late HSRs located to the site of HA injection were reported after infection with the SARS-CoV-2 in two female patients [28,29].

In addition, vaccines excipients, such as adjuvants, were also suggested as causes of adverse responses. It has been suggested that the PEG ingredients in the vaccine have a role in the mechanism of late HSRs to the mRNA vaccines; specifically in the BNT162b2v excipients, the plausible role of the PEG 2000 has been highlighted. PEG has several chemical properties that make it especially useful in various biological, chemical and pharmaceutical settings and is considered a non-toxic and non-immunogenic material which can be added to media and conjugated to molecules without interfering in cellular functions.

In fact, although HA molecules are composed of polysaccharide and mostly considered as non-immunogenic [30], many HA preparations do contain PEG and late HSRs to this compound are relatively common with an incidence of 4.2–6.7% [31,32]. The PEGs used in most HA preparations are PEG 3350 and PEG 4000, which are also frequently used in creams, shampoos, injectable medications and orally taken laxatives. This may further support the notion that prior sensitization to PEG, although not demonstrated in our cohort, may be of importance in regard to such late HSRs, and particularly as most occurred within 1–5 days following immunization [33,34,35].

Lastly and most interestingly, late skin sensitization to the BNT162b2v, although linked with late HSRs, was also observed in patients with no such reactions. In our study, four patients with immediate reactions (the control group) exhibited late skin sensitization, of which 2/4 (50%) were previously exposed to HA. Furthermore, although prior exposures to HA were not assessed in other studies, late skin sensitization to the BNT162b2v was surprisingly observed in twelve immunized patients with no clinical reactions at all following immunization, as well as among five other patients with immediate reactions [12], while in another study late skin sensitization to the same vaccine was reported among patients that were not immunized at all [36].

We speculate that late skin sensitization to intradermal skin tests with BNT162b2v may be induced by sensitization to other compounds patients have been exposed to, such as HA and many others that contain PEG and/or other ingredients, and that in some susceptible patients this may have led to the occurrence of late HSRs to this vaccine.

Our study has several limitations derived from its real-life nature, as most data on HA exposure were collected retrospectively. Thus, the exact skin filler brand or the precise time period from HA injection (as several injections may have been performed in the same patient) could not be ascertained. In addition, in our panel of skin tests for PEG-containing reagents, we used reagents with PEG 3350. We did not have medications containing PEG 2000, other than the BNT162b2v vaccine itself, available in our center. Furthermore, we did not use Elisa with a specific anti-RBD IgG (RBD is a lyophilized recombinant SARS-CoV-2 protein of the receptor binding domain) or a skin test using S protein to examine cellular immunity post vaccination, which was not available in our center at that time [37,38], and due to the lack of time during the pandemic we decided not to perform skin biopsies.

In summary, we found that late HSRs to the BNT162b2 vaccine were mild and that re-immunization with the second and third (booster) doses was safe with the addition of an anti-histamine as pre-medication and for 3–5 days after the vaccine. In agreement with prior studies, we found that direct intradermal skin testing with the BNT162b2 vaccine enabled detection of skin sensitization to this vaccine, regardless of clinical response. Skin testing did not offer additional information in risk stratification of patients with late HSRs, since all patients received additional vaccines. Nonetheless, we found a significant correlation between late HSRs and prior exposure to hyaluronic acid used as injected facial filler, which may further support the theory that prior sensitization to certain compounds predisposes individuals to late hypersensitivity reactions.

## Figures and Tables

**Table 1:** Comparison between patients with late hypersensitivity reactions and immediate hypersensitivity reactions to the BNT162b2 vaccine.

Parameters	Late Hypersensitivity Reactions (n = 17)	Immediate Hypersensitivity Reactions (n = 34)	*p* Value
Age (mean ± SD) in years	55 ± 16	52 ± 15	0.6
Gender (female)	15 (88%)	31 (92%)	1
Prior drug hypersensitivity *	8 (47%)	14 (41%)	0.92
Chronic urticaria	0 (0%)	5 (15%)	0.15
Mastocytosis	0 (0%)	0 (0%)	NR
Exposure to hyaluronic acid	7 (41%)	2 (6%)	0.004
Late skin sensitization to BNT162b2 vaccine **	9 (53%)	4 (12%)	0.003
Late skin sensitization to Depo-Medrol^®^ ***	0 (0%)	0 (0%)	NR

* History of reactions to different drugs/vaccines other than the BNT162b2 vaccine; ** induration of skin > 3 mm to ID injection of 1:100 dilution of the BNT162b2 vaccine. *** Induration of skin > 3 mm to ID injection of 1:10 dilution of Depo-Medrol^®^.

## Data Availability

Data supporting reported results is available at Sheba medical center recourses and will be presented by demand.
